# Classificação e caracterização da exposição de pescadores(as) artesanais expostos(as) ao derramamento de petróleo em Pernambuco, Brasil

**DOI:** 10.1590/0102-311XPT025425

**Published:** 2026-01-19

**Authors:** José Erivaldo Gonçalves, Verônica Maria Cadena Lima, João Pedro Ferreira Santos, Idê Gomes Dantas Gurgel, Rita de Cassia Franco Rego, Mariana Olívia Santana dos Santos

**Affiliations:** 1 Instituto Aggeu Magalhães, Fundação Oswaldo Cruz, Recife, Brasil.; 2 Instituto de Matemática e Estatística, Universidade Federal da Bahia, Salvador, Brasil.; 3 Faculdade de Medicina da Bahia, Universidade Federal da Bahia, Salvador, Brasil.; 4 Departamento de Saúde Coletiva, Universidade Federal do Rio Grande do Norte, Natal, Brasil.

**Keywords:** Exposição Ambiental, Saúde Ambiental, Poluição por Petróleo, Vigilância em Saúde do Trabalhador, Environmental Exposure, Environmental Health, Petroleum Pollution, Surveillance of the Workers Health, Exposición a Riesgos Ambientales, Salud Ambiental, Contaminación por Petróleo, Vigilancia de la Salud del Trabajador

## Abstract

Este estudo buscou classificar e caracterizar a exposição de pescadores(as) artesanais de Pernambuco, Brasil, ao petróleo durante o desastre-crime ocorrido em 2019 no litoral do Nordeste do país. Foi realizado um estudo epidemiológico transversal envolvendo entrevistas com 1.259 pescadores(as) artesanais registrados(as) em 27 colônias e/ou associações de pescadores do litoral de Pernambuco. Para a classificação do grau de exposição, utilizou-se a análise de agrupamentos com a técnica não hierárquica k-modas. A exposição ao petróleo foi classificada em três grupos: baixa (72,3%) - maioria sem contato direto; média (12,3%) - 66,4% manipularam materiais contaminados, 60% sentiram cheiro e 63% tiveram contato ocasional; e alta (15,4%) - 77,8% lidaram com resíduos, 81% sentiram forte odor e 72,2% relataram irritação na pele. Na limpeza do petróleo, dois grupos foram identificados: baixa exposição (73,1%) - 92,9% não participou; e média (26,9%) - todos participaram, 45,1% usaram instrumentos contaminados, 79,9% relataram forte odor e 20% tiveram contato frequente com petróleo. O litoral norte de Pernambuco apresentou a maior porcentagem de indivíduos no grupo de alta exposição (17,1%). Sendo este grupo, formado majoritariamente por mulheres. Os(as) pescadores(as) foram expostos(as) ao petróleo tanto no trabalho quanto na limpeza das praias, recifes e manguezais, muitos(as) com altos índices de exposição. Estes resultados exigem estratégias para o monitoramento da saúde física e mental desta população, além de avaliação de bioindicadores e ações de vigilância em saúde do trabalhador e ambiental.

## Abstract (en)

This study sought to classify and characterize the exposure of artisanal fishers from Pernambuco, Brazil, to oil during disaster-crime that took place on the Northeast coast of the country in 2019. A cross-sectional epidemiological study was conducted involving interviews with 1,259 artisanal fishers registered with 27 fishing colonies and/or associations along the coast of Pernambuco. Cluster analysis was employed with non-hierarchical k-modes for the classification of the degree of exposure: low (72.3%) - most had no direct contact; medium (12.3%) - 66.4% handled contaminated materials, 60% smelled the odor, and 63% had occasional contact; and high (15.4%) - 77.8% handled waste, 81% smelled a strong odor, and 72.2% reported skin irritation. Two exposure groups were identified for the oil cleanup activities: low (73.1%) - 92.9% did not participate; and medium (26.9%) - all participated, 45.1% used contaminated instruments, 79.9% reported a strong odor, and 20% had frequent contact with the oil. The northern coast of Pernambuco had the highest percentage of individuals in the high-exposure group (17.1%). This group was formed predominantly of women. Fishers were exposed to oil both at work and while cleaning beaches, reefs, and mangroves, many with high exposure rates. These results underscore the need for strategies to monitor the physical and mental health of this population, as well as the assessment of bioindicators and the implementation of occupational and environmental health surveillance actions.

## Abstract (es)

Este estudio buscó clasificar y caracterizar la exposición de los(as) pescadores(as) artesanales de Pernambuco, Brasil, al petróleo durante el desastre criminal ocurrido en 2019 en la costa noreste del país. Se realizó un estudio epidemiológico transversal mediante entrevistas con 1.259 pescadores(as) artesanales registrados(as) en 27 colonias y/o asociaciones de la costa de Pernambuco. Para clasificar el grado de exposición, se utilizó el análisis de agrupamientos con la técnica no jerárquica k-modas. La exposición al petróleo se clasificó en tres grupos: baja (72,3%) -la mayoría sin contacto directo; media (12,3%) -el 66,4% manipuló materiales contaminados, el 60% percibió el olor y el 63% tuvo contacto ocasional; y alta (15,4%) -el 77,8% manipuló residuos, el 81% percibió un olor fuerte y el 72,2% informó irritación cutánea. En la limpieza del petróleo, se identificaron dos grupos: baja exposición (73,1%) -el 92,9% no participó; y media (26,9%) -todos participaron, el 45,1% utilizó instrumentos contaminados, el 79,9% reportó un fuerte olor y el 20% tuvo contacto frecuente con el petróleo. La costa norte de Pernambuco presentó el mayor porcentaje de individuos en el grupo de alta exposición (17,1%). Este grupo está compuesto principalmente por mujeres. Los(as) pescadores(as) estuvieron expuestos(as) al petróleo tanto en el trabajo como en la limpieza de playas, arrecifes y manglares, muchos de ellos con altos índices de exposición. Estos resultados exigen estrategias para el seguimiento de la salud física y mental de esta población, además de la evaluación de bioindicadores y medidas de vigilancia de la salud laboral y ambiental.

## Introdução

Os casos de desastres relacionados a matrizes energéticas como petróleo têm se tornado frequentes em todo o mundo ^1^. Esses desastres socioambientais são responsáveis por diversos e profundos danos ao ambiente e à saúde individual e coletiva de comunidades atingidas ^2^ ^,^ ^3^. Constituem um desafio à saúde pública para a compreensão dos efeitos à saúde física e mental dos indivíduos expostos, pois são necessárias abordagens complexas e interdisciplinares, interseccionando conhecimentos de áreas como a toxicologia e a epidemiologia crítica ^4^ ^,^ ^5^ ^,^ ^6^.

No Brasil, o desastre-crime do derramamento de petróleo, ocorrido em 2019, afetou comunidades do litoral dos nove estados do Nordeste e Rio de Janeiro e Espírito Santo na Região Sudeste. Particularmente pescadores(as) artesanais, cujo processo de vulnerabilização socioambiental se intensificou neste contexto. É considerado um dos maiores desastres em extensão ocorrido no Atlântico Sul. Foram cerca de 5,3 mil toneladas de petróleo recolhidos em mais de 130 municípios e quase 4 mil quilômetros de extensão de costa atingida, entre rios, estuários, manguezais, unidades de conservação e camadas mais profundas do oceano ^2^ ^,^ ^7^ ^,^ ^8^. Os compostos químicos identificados no petróleo associado ao derramamento são hidrocarbonetos policíclicos aromáticos, como acenafteno, fenantreno, fluoreno, antraceno, pireno, conformando um petróleo de alta densidade ^5^.

Esta constituição incide processos de desequilíbrio e contaminação de todo ecossistema marinho e danos socioeconômicos aos(às) pescadores(as) artesanais que vivem uma relação de dependência com o território ^9^ ^,^ ^10^ ^,^ ^11^. Este evento pode ser caracterizado como um desastre-crime, implicado pela morosidade na resposta efetiva ao desastre, indefinição de responsabilidade, debilidade de ações voltadas para a proteção social, ambiental e em saúde, explicitada pelas fragilidades da gestão de risco nos processos de preparação e mitigação de danos ^12^ ^,^ ^13^.

Alguns estudos têm abordado os danos à saúde de pessoas expostas a derramamentos de petróleo, como os estudos produzidos a partir do desastre ocorrido no Golfo do México, nos Estados Unidos em 2010 ^14^, na Coreia do Sul em 2007 ^15^, e na Costa da Galícia, na Espanha em 2002 ^16^. Entretanto, ainda são incipientes as estratégias de medição de exposição dos indivíduos no contexto de desastres. A exposição aos hidrocarbonetos policíclicos aromáticos, dentre eles o benzeno, que é comprovadamente carcinogênico, independente da dose exposta, sendo um fator que implica preocupações em casos de exposição ao petróleo ^17^.

A relação entre exposição ao petróleo e adoecimento está, em muitos casos, implicada nas condições de maior exposição ao petróleo, como por exemplo, o contato direto com a pele associado a sintomas de ansiedade ^18^ ou a incidência de problemas cardiovasculares por meio da construção de índices de exposição ^14^. Classificar o nível de exposição é uma atividade imprescindível para compreensão dos danos e das relações que podem ser estabelecidas entre a exposição e os efeitos na saúde física e mental das pessoas afetadas ^19^.

No desastre-crime do derramamento do petróleo no litoral brasileiro, voluntários, principalmente pescadores(as), foram às praias para o recolhimento de material sem orientação técnica e sem equipamentos de segurança individual (EPIs) apropriados, assim como muitos(as) pescadores(as) encontraram a substância enquanto trabalhavam ^8^. Observando os possíveis efeitos da exposição, este estudo visa classificar e caracterizar esta exposição ao petróleo em pescadores(as) artesanais residentes em Pernambuco durante o desastre-crime entre agosto e novembro de 2019 no litoral do Nordeste brasileiro.

## Método

Trata-se de estudo epidemiológico de corte transversal realizado na Região Nordeste do Brasil, com dados coletados nos 16 municípios que compõem toda a costa do litoral de Pernambuco e que foram afetados pelo desastre-crime do derramamento do petróleo em 2019: Cabo de Santo Agostinho, Ipojuca, Rio Formoso, Sirinhaém, Tamandaré, Barreiros, São José da Coroa Grande, Goiana, Igarassu, Itapissuma, Ilha de Itamaracá, Recife, Jaboatão dos Guararapes, Paulista, Olinda e Abreu e Lima. O Estado de Pernambuco foi selecionado por questões logísticas e de articulação prévia com movimentos sociais da pesca artesanal no estado e dimensão do impacto, sendo o segundo estado com maior quantidade de petróleo recolhido e cerca de 70% de área litorânea atingida ^12^ ^,^ ^20^.

Foi utilizado como instrumento de pesquisa um questionário validado por meio do método Delphi e que tem como objetivo investigar os efeitos do derramamento de petróleo na saúde de pescadores(as) artesanais. O instrumento possui originalmente 325 perguntas distribuídas em 13 blocos: identificação e controle, informações gerais, situação socioeconômica e condições de vida, histórico e organização de trabalho, organização das atividades pesqueiras durante o derramamento, exposição associada à limpeza do petróleo, consumo de frutos do mar, percepção do impacto do derramamento, medidas clínicas, outras informações de saúde, estilo de vida, qualidade de vida e COVID-19 ^21^. Foi acrescido ao questionário original um bloco com variáveis relacionadas à saúde mental, constituindo, no total, 14 blocos e 361 perguntas. Para a aplicação do questionário, foi utilizado o aplicativo *offline* HCMaps, instalado em *tablets*, e a aplicação de cada questionário durou em média 60 minutos ^22^. A coleta de dados ocorreu entre setembro de 2021 a agosto de 2022.

A população do estudo foi composta por 12.472 pescadores(as), registrados nas 27 colônias e/ou associações de pescadores(as) do litoral de Pernambuco ^23^. Os critérios de inclusão dos participantes foram: indivíduos maiores de 18 anos e trabalhadores da pesca artesanal que realizam pesca em pequena escala e exerciam a atividade durante o período do desastre-crime do derramamento do petróleo. Como critério de exclusão definiu-se: trabalhadores aposentados e/ou que estavam afastados do trabalho durante o desastre-crime do derramamento do petróleo.

Para definição da amostra, o litoral pernambucano foi estratificado em três segmentos (norte, metropolitano e sul), adotando um efeito de estudo de 1,5 com prevalência de 40% para efeitos na saúde ^24^, erro de 3,5% e 95% de confiança, resultando numa amostra mínima de 1.065 indivíduos. Foi adicionado ao valor amostral as possibilidades de perdas na ordem de 25%, resultando em uma amostra de 1.331 pessoas. Como o número total de cadastrados em cada litoral era de aproximadamente 1/3 da população, foi realizada uma distribuição proporcional para cada litoral e em seguida pelas associações/colônias de cada municípios. Após a finalização do trabalho de campo, realizou-se a adequação aos critérios de inclusão e exclusão da pesquisa, obtendo-se uma amostra final de 1.259 pessoas, superando em, aproximadamente, 18% o tamanho mínimo da amostra prevista.

Para a classificação dos(as) pescadores(as) artesanais quanto ao nível de exposição, foram selecionadas 22 variáveis relacionadas à exposição ao petróleo no processo do trabalho da pesca e 17 variáveis relacionadas à exposição ao petróleo no processo de atividades voluntárias de limpeza do petróleo (Quadro 1).

**Quadro 1:** Variáveis relacionadas à exposição de pescadores(as) ao petróleo no processo de trabalho e na limpeza das praias durante o desastre-crime do petróleo em 2019. Pernambuco, Brasil.

VARIÁVEIS	CATEGORIAS
Relacionadas à exposição durante o processo de trabalho da pesca artesanal	
Você encontrou óleo/petróleo enquanto pescava/mariscava?	1. Sim 2. Não 3. Não soube responder
Quando você encontrou óleo/petróleo, você continuou pescando na área?	
Você manipulou ou reparou redes de pesca ou outros equipamentos de pesca que continham resíduos de petróleo?	
Após o contato com o petróleo os locais ficaram irritados?	
Após o contato com petróleo os locais arderam ou ficaram vermelhos?	
Você tinha algum corte ou ferida nos locais com que o petróleo do derramamento entrou em contato?	
O petróleo entrou em contato direto com a sua pele enquanto você trabalhava?	
Relacionadas à atividade voluntária de limpeza das praias	
No período do derramamento, você ajudou a retirar os resíduos de petróleo?	1. Sim 2. Não
Com que frequência você utilizou instrumentos com esses resíduos?	1. Sempre 2. Muitas vezes 3. Às vezes 4. Raramente 5. Nunca 6. Não soube responder
Relacionadas à exposição durante o processo de trabalho ou ao trabalho voluntário de limpeza das praias	
Com que frequência, em média, o petróleo entrou em contato direto com sua cabeça?	1. Sempre 2. Muitas vezes 3. Às vezes 4. Raramente 5. Nunca 6. Não soube responder
Com que frequência, em média, o petróleo entrou em contato direto com seus olhos?	
Com que frequência, em média, o petróleo entrou em contato direto com sua boca?	
Com que frequência, em média, o petróleo entrou em contato direto com braços?	
Com que frequência, em média, o petróleo entrou em contato direto com seu tórax?	
Com que frequência, em média, o petróleo entrou em contato direto com suas costas?	
Com que frequência, em média, o petróleo entrou em contato direto com suas mãos?	
Com que frequência, em média, o petróleo entrou em contato direto com sua coxa?	
Com que frequência, em média, o petróleo entrou em contato direto com sua perna?	
Com que frequência, em média, o petróleo entrou em contato direto com seus pés?	
Em média, com que frequência você sentiu o cheiro enquanto retirava o petróleo?	
O petróleo deixou marcas visíveis em seu corpo?	1. Sim 2. Não 3. Não soube responder
Quando o odor estava presente, como você classificaria a intensidade do odor?	1. Muito forte 2. Forte 3. Moderado 4. Leve 5. Muito leve 6. Não sentiu
Classifique a intensidade que o odor foi irritante para os olhos	
Classifique a intensidade que o odor foi irritante para o nariz	

Fonte: elaboração própria.

Após a seleção das variáveis, os dados foram recodificados: 0 = “não” ou “não soube responder” e 1 = “sim”. As variáveis de intensidade e frequência à exposição que estão em escala Likert foram categorizadas em: 0 = “não sentiu”; 1 = “muito leve”, “leve” ou “moderado”; 2 = “forte” ou “muito forte”. Outras variáveis de frequência a exposição em escala Likert foram categorizadas em: 0 = “nunca” ou “não soube responder”; 1 = “às vezes” ou “raramente”; 2 = “muitas vezes” ou “sempre”.

Importante destacar que a categoria “não soube responder” apresentou baixa frequência, variando de 0% a 2,3% para a maioria das variáveis. À exceção das variáveis “Você manipulou ou reparou redes de pesca ou outros equipamentos de pesca que continham resíduos de petróleo?” e “O petróleo entrou em contato direto com a sua pele enquanto você trabalhava?”, que apresentaram percentuais variando em torno de 7% a 8%. Por simplicidade, foi considerado que a resposta “não soube responder” denota pouca importância ao derramamento de petróleo por parte do pescador artesanal, sendo, portanto, acrescida às categorias “não” e “nunca”.

O k-modas é uma extensão do k-médias utilizado para dados quantitativos. No método k-modas, a estatística utilizada é a moda e, a cada passo do algoritmo, a moda de cada grupo é atualizada. Para a comparação entre os indivíduos, foi adotada uma medida de dissimilaridade baseada no coeficiente de concordância simples. Quanto menor os valores de dissimilaridade, mais similares serão os elementos que estão sendo comparados ^25^. Para leitura e análise dos dados, utilizou-se a plataforma computacional R, versão 4.2.1 (https://cran.r-project.org/). Esta pesquisa foi aprovada pelo Comitê de Ética e Pesquisa do Instituto Aggeu Magalhães, Fundação Oswaldo Cruz (CAAE: 25398119.9.0000.5190) e seguiu as diretrizes da *Resolução nº 446/2012* da Comissão Nacional de Ética em Pesquisa.

## Resultados

O resultado da análise de agrupamento permitiu classificar os(as) pescadores(as) artesanais, em relação ao processo de trabalho, em três grupos: baixa, média e alta exposição. O grupo de baixa exposição é composto por 910 (72,3%) indivíduos. Em geral, este grupo é composto por indivíduos cuja maioria (82,2%) não manipulou ou reparou redes de pesca ou outros equipamentos de pesca que continham resíduos de petróleo, 57% não sentiu o cheiro do petróleo e pelo menos 90,3% relatam que o petróleo não entrou em contato com a pele ou parte do corpo.

O grupo de média exposição é composto de 155 (12,3%) indivíduos. Neste grupo, 66,4% manipularam ou repararam redes de pesca ou outros equipamentos de pesca que continham resíduos de petróleo; 60% sentiram cheiro de petróleo ocasionalmente; e aproximadamente 58% classificaram a intensidade do odor como forte; em torno de 63% relatou que o petróleo entrou em contato com as mãos e pés de modo ocasional; e 60% relataram ardência ou vermelhidão na pele após o contato com petróleo.

O grupo de alta exposição é formado por 194 (15,4%) indivíduos. Neste grupo, observam-se os maiores níveis de exposição: 77,8% relatou que manipulou ou reparou redes de pesca ou outros equipamentos de pesca que continham resíduos de petróleo e que sentiu o cheiro de petróleo; aproximadamente 81% classificou a intensidade do odor como forte ou muito forte; a maioria relatou forte irritação ao nariz 59,2% ou olhos 53,6% mediante a intensidade do cheiro do petróleo; a maioria relatou contato do petróleo com alguma parte do corpo (braços: 61,9%; mãos: 77,3%; perna: 66%; pés: 77,8%) e 72,2% relatou ardência ou vermelhidão na pele após o contato com petróleo (Tabela 1).

**Tabela 1:** Características dos *clusters* de baixa, média e alta exposição de pescadores(as) ao petróleo durante o trabalho da pesca artesanal. Pernambuco, Brasil.

Questões	***Clusters* de exposição (%)**		
	Baixa	Média	Alta
Você encontrou óleo/petróleo enquanto pescava?			
Não	42,4	0,0	0,0
Sim	57,6	100,0	100,0
Quando você encontrou petróleo, você continuou pescando na área?			
Não	80,0	63,9	61,3
Sim	20,0	36,1	38,7
Você manipulou ou reparou redes de pesca ou outros equipamentos de pesca que continham resíduos de petróleo?			
Não	82,2	33,6	22,2
Sim	17,8	66,4	77,8
Com que frequência você sentiu esse cheiro de petróleo?			
Nunca	57,0	7,7	2,1
Ocasionalmente	21,8	60,0	20,1
Sempre	21,2	32,3	77,8
Como você classificaria a intensidade do odor/cheiro?			
Não sentiu	57,2	7,7	2,6
Moderado	17,1	34,2	16,5
Forte	25,6	58,1	80,9
Classifique a intensidade que o odor/cheiro foi irritante para os olhos			
Não sentiu	80,7	69,6	26,2
Moderado	10,2	21,9	20,1
Forte	9,01	8,3	53,6
Classifique a intensidade que o odor/cheiro foi irritante para o nariz			
Não sentiu	75,7	56,7	22,6
Moderado	12,0	25,1	18,0
Forte	12,2	18,0	59,2
O petróleo entrou em contato direto com a sua pele enquanto você trabalhava?			
Não	90,3	0,0	0,0
Quantas vezes o petróleo entrou em contato com sua cabeça?			
Nunca	99,8	90,3	77,8
Ocasionalmente	0,1	9,0	6,7
Sempre	0,0	0,64	15,4
Quantas vezes o petróleo entrou em contato com seus olhos?			
Nunca	100,0	93,5	82,4
Ocasionalmente	0,0	6,4	5,6
Sempre	0,0	0,0	11,8
Quantas vezes o petróleo entrou em contato com sua boca?			
Nunca	100,0	94,8	86,1
Ocasionalmente	0,0	4,5	3,6
Sempre	0,0	0,6	10,3
Quantas vezes o petróleo entrou em contato com seus braços?			
Nunca	98,4	67,1	29,9
Ocasionalmente	1,1	28,3	8,2
Sempre	0,4	4,5	61,9
Quantas vezes o petróleo entrou em contato com seu tórax?			
Nunca	100,0	90,3	70,1
Ocasionalmente	0,0	9,6	5,1
Sempre	0,0	0,0	24,7
Quantas vezes o petróleo entrou em contato com suas costas?			
Nunca	100,0	92,2	73,7
Ocasionalmente	0,0	7,7	5,1
Sempre	0,0	0,0	21,1
Quantas vezes o petróleo entrou em contato com suas mãos?			
Nunca	95,4	21,9	17,0
Ocasionalmente	2,1	63,9	5,6
Sempre	2,4	14,2	77,3
Quantas vezes o petróleo entrou em contato com sua coxa?			
Nunca	99,6	80,0	55,1
Ocasionalmente	0,3	18,7	6,1
Sempre	0,0	1,2	38,6
Quantas vezes o petróleo entrou em contato com sua perna?			
Nunca	98,5	60,6	24,7
Ocasionalmente	1,1	36,1	09,3
Sempre	0,3	3,2	66,0
Quantas vezes o petróleo entrou em contato com seus pés?			
Nunca	95,2	27,1	15,4
Ocasionalmente	2,6	63,2	6,7
Sempre	2,1	09,6	77,8
Após o contato com o petróleo os locais ficaram irritados?			
Não	99,5	42,6	25,7
Sim	0,4	57,4	74,2
Após o contato com petróleo os locais arderam ou ficaram vermelhos?			
Não	99,2	40,0	27,8
Sim	0,7	60,0	72,2
Você tinha algum corte ou ferida nos locais com que o petróleo do derramamento entrou em contato?			
Não	99,4	84,5	83,5
Sim	0,6	15,4	16,4
O contato petróleo deixou marcas visíveis em seu corpo?			
Não	99,3	85,8	81,9
Sim	0,7	14,2	18,1

Fonte: elaboração própria.

Nota: moderado = “muito leve, leve ou moderado”; forte = “forte ou muito forte”; ocasionalmente = “raramente ou às vezes”; sempre = “muitas vezes ou sempre”.

No caso dos(as) pescadores(as) artesanais envolvidos no trabalho de limpeza do petróleo, a análise de agrupamento classificou estes indivíduos em dois grupos, um de baixa e outro de média exposição. O grupo de baixa exposição é composto por 920 (73,1%) indivíduos. Em geral, este grupo não participou do processo de limpeza do petróleo, 92,9%, apresentando uma baixa frequência em relação às categorias do estudo que se referem a uma exposição mais aguda como “sempre, muitas vezes” ou “forte, muito forte”.

O grupo de média exposição é composto por 339 (26,9%) indivíduos. Neste grupo, todos os indivíduos participaram da limpeza do petróleo e 45,1% deles utilizaram instrumentos com resíduos de petróleo. A maioria das pessoas (79,9%) classificaram o odor como “forte ou muito forte”, 41% classificaram como irritante para o nariz e 33,3% como irritante para os olhos. Neste grupo, pelo menos 1/5 dos indivíduos relataram ter havido contato do petróleo com mãos, braços, pés e pernas. A maioria relatou sentir o cheiro de petróleo (81,12%) (Tabela 2).

**Tabela 2:** Características dos *clusters* de baixa e média exposição de pescadores(as) ao petróleo durante o trabalho de limpeza do petróleo. Pernambuco, Brasil.

Questões	***Clusters* de exposição (%)**	
	Baixa	Média
No período do derramamento, você ajudou a retirar os resíduos de petróleo?		
Não	92,9	0,0
Sim	7,1	100,0
Frequência, em média, com que o petróleo entrou em contato direto com sua cabeça?		
Nunca	100,0	92,0
Ocasionalmente	0,0	3,8
Sempre	0,0	4,1
Frequência, em média, com que o petróleo entrou em contato direto com seus olhos?		
Nunca	99,8	94,6
Ocasionalmente	0,0	2,6
Sempre	0,1	2,6
Frequência, em média, com que o petróleo entrou em contato direto com sua boca?		
Nunca	100,0	94,4
Ocasionalmente	0,0	2,3
Sempre	0,0	3,2
Frequência, em média, com que o petróleo entrou em contato direto com braços?		
Nunca	98,9	70,8
Ocasionalmente	0,5	8,5
Sempre	0,5	20,6
Frequência, em média, com que o petróleo entrou em contato direto com seu tórax?		
Nunca	99,8	90,5
Ocasionalmente	0,1	2,3
Sempre	0,0	7,0
Frequência, em média, com que o petróleo entrou em contato direto com suas costas?		
Nunca	99,8	91,7
Ocasionalmente	0,1	2,6
Sempre	0,0	5,6
Frequência, em média, com que o petróleo entrou em contato direto com suas mãos?		
Nunca	98,2	56,0
Ocasionalmente	0,7	13,2
Sempre	0,9	30,6
Frequência, em média, com que o petróleo entrou em contato direto com sua coxa?		
Nunca	99,6	83,7
Ocasionalmente	0,1	3,8
Sempre	0,2	12,3
Frequência, em média, com que o petróleo entrou em contato direto com sua perna?		
Nunca	99,2	68,7
Ocasionalmente	0,4	7,6
Sempre	0,3	23,6
Frequência, em média, com que o petróleo entrou em contato direto com seus pés?		
Nunca	97,8	56,6
Ocasionalmente	0,8	11,5
Sempre	1,3	31,8
O petróleo deixou marcas visíveis em seu corpo?		
Não	99,7	92,0
Sim	0,2	7,9
Em média, com que frequência você sentiu o cheiro enquanto retirava o petróleo?		
Sempre	97,1	0,5
Nunca	2,2	18,2
Ocasionalmente	0,54	81,1
Quando o odor estava presente, como você classificaria a intensidade do odor?		
Não sentiu	96,7	0,3
Moderado	3,0	19,7
Forte	0,2	79,9
Classifique a intensidade que o odor foi irritante para os olhos		
Não sentiu	99,5	45,1
Moderado	0,4	21,5
Forte	0,0	33,3
Classifique a intensidade que o odor foi irritante para o nariz		
Não sentiu	99,3	33,6
Moderado	0,6	25,3
Forte	0,0	41,0
Com que frequência você utilizou instrumentos com esses resíduos?		
Nunca	97,8	41,5
Ocasionalmente	1,4	13,2
Sempre	0,7	45,1

Fonte: elaboração própria.

Nota: moderado = “muito leve, leve ou moderado”; forte = “forte ou muito forte”; ocasionalmente = “raramente ou às vezes”; sempre = “muitas vezes ou sempre”.

A denominação da classificação dos grupos na remoção em categorias de baixa e média exposição deveu-se à comparação dos indivíduos expostos durante o trabalho da pesca e da remoção, em que se destaca uma frequência elevada nas variáveis de intensidade para a pesca relacionadas ao grupo de alta exposição. Além disso, observa-se que, no contato do petróleo com partes do corpo, há diferenças percentuais em relação a uma maior exposição durante o processo de trabalho da pesca, em detrimento dos indivíduos que participaram da remoção voluntária do petróleo.

Considerando a distribuição da exposição durante o trabalho da pesca por sexo e no litoral, observa-se que o litoral norte possui a maior percentagem de indivíduos classificados no grupo de alta exposição (17,1%), seguido do litoral sul (16,5%). É possível observar que em todas as regiões, o grupo de alta exposição é formado na maioria por mulheres, com destaque para os litorais norte (13,3%) e metropolitano (8,3%). O teste qui-quadrado indica que não há associação entre o nível de exposição e sexo, para todas as localidades (Tabela 3).

**Tabela 3:** Classificação dos pescadores quanto ao nível de exposição durante o trabalho da pesca e na remoção de petróleo por sexo e localidade. Pernambuco, Brasil.

Localidade/Nível de exposição	Masculino	Feminino	Total	Valor de p *
	n (%)	n (%)	n (%)	
Classificação da exposição ao petróleo durante o trabalho da pesca				
Litoral metropolitano				
Baixa	106 (24,3)	216 (49,6)	322 (73,9)	0,753
Média	22 (5,1)	36 (8,3)	58 (13,3)	
Alta	19 (4,3)	36 (8,3)	55 (12,6)	
Litoral norte				
Baixa	85 (18,3)	242 (51,9)	327 (70,2)	0,699
Média	13 (2,8)	46 (9,8)	59 (12,6)	
Alta	18 (3,8)	62 (13,3)	80 (17,1)	
Litoral sul				
Baixa	98 (27,3)	163 (45,5)	261 (72,8)	0,217
Média	17 (4,7)	21 (5,9)	38 (10,6)	
Alta	29 (8,1)	30 (8,4)	59 (16,5)	
Classificação da exposição ao petróleo durante a atividade de remoção				
Litoral metropolitano				
Baixa	111 (25,5)	207 (47,5)	318 (73,0)	0,487
Média	36 (8,2)	81 (18,6)	117 (26,8)	
Litoral norte				
Baixa	82 (17,6)	255 (54,7)	337 (72,3)	0,740
Média	34 (7,3)	95 (20,4)	129 (27,3)	
Litoral sul				
Baixa	101 (28,2)	164 (45,8)	265 (74,0)	0,211
Média	43 (12,1)	50 (13,9)	93 (26,0)	

* Teste qui-quadrado de associação.

Em relação às atividades de limpeza do petróleo, observam-se percentuais similares do grupo de maior exposição por litoral. O litoral norte (27,3%) e o litoral metropolitano (26,8%) se apresentam como as regiões que agrupam a maioria das pessoas classificadas enquanto grupo de média exposição. Este cenário se repete em relação ao sexo, uma vez que, embora haja predomínio do sexo feminino em todas as regiões, evidencia-se percentual mais elevado nos litorais norte (20,4%) e metropolitano (18,6%) (Tabela 3).

## Discussão

Nos anos de 2000 e 2019, foram registrados mais de sete mil grandes desastres, a maioria relacionados às mudanças climáticas extremas, o que representa o dobro em relação ao século passado (1980/1999) ^1^. Um aumento mundial no número de desastres naturais e tecnológicos afetou cerca de quatro bilhões de pessoas no mundo e causou prejuízos superiores a 1,5 trilhão de dólares ^1^. No entanto, é importante destacar que desastres tecnológicos, como os envolvendo derramamentos de petróleo, possuem características distintas. Santos et al. ^2^ sistematizaram os principais desastres com petróleo no mundo, apontando a diminuição do tempo de sua ocorrência e aumento dos desastres, gerando impactos nos sistemas e serviços de saúde.

A exposição humana aos hidrocarbonetos aromáticos policíclicos, como benzeno, xileno e tolueno, está relacionada a uma via dupla de metabolização resultando numa condição de alta metabolização do composto no organismo humano mesmo em pequenas doses de exposição, acarretando efeitos nocivos ^26^. Para esta discussão, é importante considerar uma toxicologia crítica que compreenda mesmo os menores níveis de exposição como um perigo à saúde e a inexistência de um limiar seguro de exposição a compostos químicos, considerando os efeitos que podem ocorrer a médio e longo prazo ^19^.

Nos resultados deste estudo, pode-se identificar que 4,6% dos indivíduos pesquisados têm uma dupla exposição que se apresenta num primeiro momento na exposição durante o trabalho da pesca e depois durante a limpeza do petróleo. De acordo com Gonçalves et al. ^27^, no período de 1-3 meses após o derramamento de petróleo, os(as) pescadores(as) artesanais relataram sintomas neurológicos e respiratórios, os quais podem estar associados à exposição durante o desastre-crime do derramamento de petróleo em 2019.

Em casos de desastres por derramamentos de petróleo, como os ocorridos em 2010 no Golfo do México, após a explosão da plataforma Deepwater Horizon, em 2007 na Coreia do Sul, e em 2002 na Espanha, os estudos mostraram que os efeitos na saúde estão relacionados principalmente aos órgãos e sistemas: neurológico, cardiovascular, respiratório e pele/mucosas ^28^. Observando os grupos de exposição durante o trabalho da pesca e da limpeza do petróleo, pode-se refletir sobre a dinâmica do trabalho da pesca que expõem o corpo de forma direta ao ambiente aquático, contaminado com petróleo, por este fato, percebe-se diferenças nas frequências da área de contato com o petróleo quando comparado a atividade dos indivíduos expostos durante a remoção.

A contaminação ambiental por petróleo é um processo que se estende para além dos impactos imediatos dos desastres como ocorrido no Brasil em 2019. Esses processos, incluindo a adsorção de óleo por material particulado em suspensão e eventual deposição de óleo no fundo do mar, são fatores cruciais que influenciam o destino e os efeitos do petróleo em habitats marinhos tropicais e consequentemente na saúde ambiental das comunidades. A estrutura complexa e a lenta taxa de biodegradação do petróleo, juntamente com o potencial de biomagnificação, levantam preocupações sobre sua inclusão em estudos de poluição ^29^. No caso do derramamento de petróleo no Brasil, os efeitos ecotoxicológicos no oceano foram observados a longa distância do ponto de liberação ^11^ ^,^ ^30^.

Müller et al. ^11^ apresentaram resultados sobre o branqueamento de corais na região da Área de Preservação Ambiental (APA) Costa dos Corais, Tamandaré (Pernambuco), ao longo do tempo e alertam para os fatores que podem potencializar a mortalidade de corais como os estresses preexistentes, poluição, plásticos e, no caso do Nordeste, poluição pelo derramamento de petróleo em 2019. Outros sistemas, como manguezais, estuários, rios e recifes de corais, também foram afetados. Ressalte-se que não se sabe exatamente a extensão dos danos ambientais, podendo estes serem agudos ou crônicos, exigindo monitoramento e estudos a curto, médio e longo prazo ^31^. Os danos ambientais reverberam na manutenção da biodiversidade e da vida no ecossistema marinho que podem alterar relações simbióticas e comprometer a biodisponibilidade de peixes. Interferindo a médio e longo prazo na economia pesqueira de pessoas que dependem desse tipo de trabalho e na exposição a compostos tóxicos contínua atrelada diretamente ao modo de vida dessas comunidades ^11^.

O processo de monitoramento dos indivíduos expostos é fundamental e demanda uma vigilância em saúde, do trabalhador e ambiental que compreenda a complexidade dos processos de exposição a partir da concepção de desastres ampliados e a uma saúde crítica, num diálogo interdisciplinar entre o campo da saúde coletiva e a saúde ambiental ^32^ ^,^ ^33^. O recorte social da exposição infere questões sobre o impacto da exposição em si e as condições circunstanciais para a exposição que estão relacionadas ao modo de vida, aos processos de subsistência, os processos de injustiças e racismo ambiental e a ineficiência de planos de contingência e atuação da gestão e da esfera política ^34^ ^,^ ^35^ ^,^ ^36^.

Durante o trabalho da pesca, 36,1% e 38,7% dos indivíduos nos grupos de média e alta exposição, respectivamente, continuaram a trabalhar mesmo após encontrar petróleo nas áreas de pesca. Este dado implica não no desconhecimento total dos impactos do petróleo na saúde, mas na necessidade da sobrevivência dessas pessoas que têm no trabalho da pesca o seu principal meio de subsistência ^27^.

No caso dos indivíduos expostos durante as atividades de limpeza do petróleo, 100% das pessoas classificadas no grupo de média exposição participaram da remoção, num impulso de defesa territorial, mas sustentado pela ausência dos órgãos competentes e responsáveis a atuarem em situações de desastres como a vivenciada por essas comunidades em 2019 em todo o Nordeste e parte do Sudeste. A morosidade na resposta por parte do Estado direcionou esses(as) pescadores(as) e voluntários(as) sem treinamento ou conhecimento técnico para remoção do petróleo nas praias e ambientes contaminados, podendo ter como consequência uma maior exposição ao petróleo ^37^.

Esses processos compõem uma estrutura de racismo e injustiças socioambientais vivenciadas pelas comunidades da pesca artesanal no Brasil, acentuadas em momentos como o desastre-crime do derramamento de petróleo em 2019 ou a pandemia da COVID-19 ^34^. É necessário compreender a exposição e suas interfaces, desprendendo-se do marco temporal linear, compreendendo a existência de processos de vulnerabilização que antecedem e que podem promover a intensificação da exposição, o momento presente da exposição e os impactos futuros, interseccionando o processo histórico, social e de trabalho dessas comunidades, à saúde e o ambiente ^34^ ^,^ ^38^.

A vigilância em saúde, particularmente relacionada à saúde do trabalhador, tem um papel fundamental no acompanhamento e monitoramento desses indivíduos, ampliando as práticas associadas à identificação de alterações metabólicas, sinais e sintomas que poderiam estar relacionados à exposição. Visto que os efeitos da exposição podem ocorrer anos após o evento e a contaminação do ambiente pode gerar uma cadeia biológica e química de exposição, principalmente associado ao processo de trabalho das comunidades da pesca artesanal que vivem na costa ^39^. Demanda-se dos serviços de saúde uma interdisciplinaridade alinhada a uma atuação territorializada que integre as condições de vida das comunidades da pesca artesanal nas matrizes de decisões.

## Considerações finais

Os(as) pescadores(as) foram expostos em diferentes níveis durante o processo de trabalho da pesca e na limpeza de petróleo das praias, recifes e mangues e, alguns em ambos os casos, com altos índices de exposição, resultando numa exposição acumulada. Pescadores(as) continuaram a trabalhar mesmo após encontrar petróleo no ambiente, este fato requer estratégias de monitoramento e vigilância em saúde do trabalhador e ambiental, para identificar e acompanhar efeitos na saúde física e mental dessas comunidades afetadas. A avaliação de bioindicadores, por exemplo, é indicado nesses casos para acompanhar alterações metabólicas e/ou fisiológicas.

Não se sabe exatamente quais e quantas são ou serão as implicações desse processo de exposição na saúde dessas comunidades ao longo dos anos, reforçando a necessidade de acompanhamento dessas pessoas pelo Sistema Único de Saúde (SUS). No entanto, em outros desastres com petróleo no mundo, as repercussões estão associadas a problemas respiratórios, neurológicos, endócrinos, cardiovasculares, partos prematuros e inclusive a diminuição significativa do consumo e venda de pescado pelas comunidades.

Nesta perspectiva, fazem-se necessárias pesquisas epidemiológicas que correlacionam grupos mais e menos expostos com alterações metabólicas e sintomatologias autorreferidas, bem como estudos de coorte para acompanhamento da evolução desses indivíduos. Este estudo pode ser utilizado como um estudo de base, dadas as medidas limítrofes a um estudo transversal, demarcado por um recorte temporal de cerca de dois anos após o desastre ocorrido em razão do cenário pandêmico da COVID-19.
